# Characterization of Extended Spectrum Cephalosporin-Resistant *Escherichia coli* Strains Isolated from Raw Poultry Carcasses in Catering Services in Northern Greece

**DOI:** 10.3390/vetsci10080487

**Published:** 2023-07-27

**Authors:** Vangelis Economou, Georgios Delis, Dimitra Stavrou, Panagiota Gousia, Anestis Tsitsos, Tilemachos Mantzios, Eirini Chouliara, Nikolaos Kolovos, Nikolaos Soultos

**Affiliations:** 1 Laboratory of Animal Food Products Hygiene and Veterinary Public Health, School of Veterinary Medicine, Aristotle University of Thessaloniki, 541 24 Thessaloniki, Greece; tsitanes@vet.auth.gr (A.T.); nkolovos872@gmail.com (N.K.); soultos@vet.auth.gr (N.S.); 2 Laboratory of Pharmacology, School of Veterinary Medicine, Aristotle University of Thessaloniki, 541 24 Thessaloniki, Greece; delis@vet.auth.gr; 3 Hellenic Army Biological Research Center, 152 36 Athens, Greece; demivet@windowslive.com; 4 Research Laboratories of Thessaloniki, Department of Food Testing, Hellenic Food Authority, 570 01 Thermi, Greece; pgousia@efet.gr; 5 Unit of Avian Medicine, Clinic of Farm Animals, School of Veterinary Medicine, Aristotle University of Thessaloniki, 546 27 Thessaloniki, Greece; mantzios@vet.auth.gr; 6 Laboratory of Technology of Food of Animal Origin, School of Veterinary Medicine, Aristotle University of Thessaloniki, 541 24 Thessaloniki, Greece; echoulia@vet.auth.gr

**Keywords:** *Escherichia coli*, poultry carcass, meat, antibiotic resistance, ESBL, AmpC, Greece

## Abstract

**Simple Summary:**

Antimicrobial resistance is a phenomenon spreading through animals and humans, often with food as a vector. In this study, the presence of extended-spectrum β-lactamase producing *E. coli* isolates was examined in raw poultry carcasses from Greece. Among the samples, 64% were positive. One hundred and twenty strains were isolated, among which 71.67% were classified as true ESBL, 20.00% as AmpC, and 8.33% as “of unknown etiology”. The genetic background of the isolates for ESBL production featured the presence of variants of the *bla*_CTX-M_ and the *bla*_TEM_ genes, with non-gene harboring strains also isolated. The results demonstrate the existence of *E. coli* isolates producing extended-spectrum β-lactamases in raw poultry meat from Greece, posing a risk for antibiotic resistance transfer to humans. Further studies are needed to access microbial resistance trends, elucidate possible transmission routes, and further strengthen public health surveillance.

**Abstract:**

Antimicrobial resistance is considered a topic of utmost interest under the concept of “One Health”, having severe implications in both human and veterinary medicine. Among the antibiotic-resistant bacteria, gram-negative bacteria, especially those belonging to the order of Enterobacterales (such as *Escherichia coli*), hold a prominent position in terms of both virulence and possessing/disseminating antimicrobial resistance (AMR) traits. The aim of this study was to examine the presence of extended-spectrum β-lactamase producing *E. coli* isolates in raw poultry carcasses collected from a university club. Five hundred raw poultry skin samples were collected from the Aristotle University of Thessaloniki (AUTh) club in Thessaloniki, Greece. A total of 64% of the samples were positive for the presence of extended-spectrum β-lactamase (ESBL)-producing *E. coli*. The isolates were further examined for their susceptibility to selected antibiotics by the disc diffusion method and were characterized as true ESBL, as producing class C cephalosporinases (AmpC) or “of unknown etiology” by the combination disc test. The 86 of the 120 isolates (71.67%) were classified as true ESBL, 24 (20.00%) as AmpC, and 10 (8.33%) as “of unknown etiology”. The isolates were screened for the occurrence of β-lactamase genes (*bla*_TEM_, *bla*_CTX-M_, *bla*_SHV,_ and *bla*_OXA_). Thirty-six isolates (32 ESBL- and 4 AmpC-phenotype) harbored both *bla*_TEM_ and *bla*_CTX-M_ genes, twenty-two isolates (among which 19 ESBL-phenotype and 2 AmpC-phenotype) harbored *bla*_CTX-M_ only, whereas twenty-six (14 ESBL- and 12 AmpC-phenotype) isolates harbored *bla*_TEM_ alone. No isolate harboring *bla*_SHV_ or *bla*_OXA_ was detected. The results demonstrate the existence of *E. coli* isolates producing extended-spectrum β-lactamases in poultry carcasses from Greece, pausing a risk for antibiotic resistance transfer to humans.

## 1. Introduction

The emergence of antimicrobial-resistant (AMR) bacteria is one of the major emerging health threats to human populations worldwide [1,2], as it has been linked to increased morbidity and mortality, as well as to rising healthcare costs [2,3]. This phenomenon is not new; the occurrence of resistance has risen along with the spread of antibiotics in both pathogenic and commensal bacteria [4]. More specifically, Enterobacterales are often considered microorganisms associated with antibiotic resistance due to several factors, including their role as both commensals and pathogens [5,6]. Among the resistance factors, the production of β-lactamases has been given much attention since they can exert resistance to β-lactam antibiotics, such as penicillins, cephalosporins, cephamycins, monobactams, and carbapenems [6]. Their importance is such that WHO has set as critical the priority of development of new antibiotics against carbapenem- and 3rd/4th generation cephalosporin-resistant Enterobacterales [7]. 

Acquired antibiotic resistance can be a consequence of the use and abuse of antibiotics [3]. Antibiotic use in human medicine has been mainly implicated in the emergence of resistance in nosocomial settings, being notorious for the occurrence of almost untreatable bacteria. Still, farm animals and their products are considered a significant pool of resistance [8]. In the era where antibiotics were used as growth promoters or for mere prevention purposes (i.e., without treatment claims), the emergence of resistant bacteria was quite common [4]. Although the use of antibiotics as growth promoters has been banned in the European Union and elsewhere, the situation is uneven in different parts of the world [9]. Among farm animals, poultry and their products are considered the most frequent carriers of ESBL- and AmpC-(collectively referred to as “extended-spectrum cephalosporinases”—ESCs) producing *Escherichia coli* [10]. Poultry farming is the most intensive form of animal production, making poultry prone to immune deficiency and disease. In addition, the large population densities facilitate the spread of bacteria within the flocks. Moreover, ESBL- and AmpC-producing Enterobacterales originating from poultry have been documented to cause infections in humans [11,12].

Many Mediterranean countries have been associated with the emergence of several new ESBL or AmpC genes [6]. The circulating antibiotic resistance factors in Greek patients follow the same trend. Nevertheless, little information is available concerning the occurrence of ESBL- and AmpC-producing bacteria in Greek poultry products. Therefore, the scope of this study was to evaluate the occurrence of ESC-producing *E. coli* in poultry products and to estimate the risk of food-borne antibiotic resistance in humans. 

## 2. Materials and Methods

### 2.1. Sample Collection 

An overall of 500 whole poultry carcasses were sampled, by collecting neck skin specimens, according to the Commission Regulation (EC) 2073/2005 on microbiological criteria for foodstuffs. The samples were procured from the university club of Aristotle University of Thessaloniki (AUTh-UC), Greece, according to its daily routine. AUTh-UC is able to provide up to 15,000 meals per day served primarily in two main refectories, which seat 1000 and 500 students, respectively. The collection of the neck skins was performed aseptically from individually packed chicken carcasses with the use of sterile utensils. All chicken carcasses originated from Greek farms and were collected randomly throughout the year on the premises of AUTh-UC. The samples were transported to the Laboratory of Animal Food Products Hygiene and Veterinary Public Health into portable coolers and processed within 4 h after collection. Samples were pooled in groups of five, all originating from the same producer and production date, resulting in one hundred pooled samples further used for examination. 

### 2.2. Microbiological Examination

The isolation procedure was aimed at selecting only resistant bacteria from the samples examined. For the isolation of resistant *E. coli*, the methods of Agersø et al. [13] and Egervärn et al. [14] were used, with modifications. The samples were rinsed with McConkey broth (Oxoid, Basingstoke, UK) supplemented with 1 mg/L cefotaxime (cefotaxime sodium salt, Sigma-Aldrich, Saint Louis, MO, USA), which also served as a pre-enrichment medium. The rinsates were incubated at 42 °C for 18 h. After enrichment, plates of Violet Red Bile Glucose agar (Oxoid, Basingstoke, UK) with 1 mg/L cefotaxime (Sigma Aldrich, Saint Louis, USA) and Chromocult TBX agar (VRBG, Merck GmbH, Darmstadt, Germany) with 1 mg/L cefotaxime were inoculated and were further incubated at 42 °C for 24 h. *E. coli* form blue to green colonies in Chromocult TBX agar and purple to pink colonies with or without halos in VRBG agar. A maximum of five characteristic colonies of each plate were picked and pure-cultured in appropriate media. An initial indole test was performed (Kovac’s reagent, Liofilchem, Roseto degli Abruzzi, Italy) followed by standard biochemical tests for isolate typing to the species level, including typing of the isolates with the use of the automated microbial identification system VITEK^®^ 2 Compact (bioMérieux, Marcy-l’Étoile, France) and the appropriate VITEK^®^ 2 GN cards (bioMérieux, Marcy-l’Étoile, France) for the biochemical identification of gram-negative strains.

### 2.3. Determination of the Susceptibility of Isolates to Antibiotics

The disc diffusion method was used for the determination of the susceptibility of isolated strains to selected antibiotics, according to the recommendations of the Clinical and Laboratory Standards Institute [15]. One or two pure colonies were picked from an overnight culture and were suspended in 10 mL of sterile normal saline. The turbidity of the suspension was adjusted to 0.5 McFarland scale with the use of a nephelometer (Densitomat, bioMérieux, France), corresponding to approximately 10^8^ CFU/mL, and an aliquot was streaked subsequently over Mueller Hinton agar plates with a sterile swab (bioMérieux, Marcy-l’Étoile, France). The antibiotic discs utilized contained penicillins [ampicillin (AMP 10 μg) and the amoxicillin-clavulanic acid combination (AMC 20/10 μg)], cephalosporins [cefotaxime (CTX 5 μg), and ceftazidime (CAZ 10 μg)], carbapenems [meropenem (MEM 10µg)], fluoroquinolones [ciprofloxacin (CIP 5 μg)], aminoglycosides [tobramycin (TOB 10µg), amikacin (AK 30µg), and gentamicin (CN 10 μg)], sulfonamides [trimethoprim-sulfamethoxazole combination (SXT 1.25/23.75 μg)], phenicols [chloramphenicol (CAF 30 μg)], and tetracyclines [tigecycline (TGC 15 μg)]. All antibiotic discs were supplied by Oxoid (Basingstoke, UK). The *E. coli* ATCC 25922 type culture was used for quality control. Inhibition zones were measured to the nearest millimeter (mm) using a digital caliper (Powerfix, Model Z22855, London, UK) and were characterized according to the EUCAST clinical resistance breakpoints [16], as mentioned in the European Commission Decision 652/2013.

### 2.4. Combination Disk Test 

For the phenotypic characterization of the isolates, the combination disk test was utilized according to CLSI [15] and EUCAST [17] guidelines. In brief, an initial inoculum was prepared as already described, and Mueller Hinton agar plates (bioMérieux, France) were inoculated. Antibiotic discs (Himedia, Mumbai, India) containing cefepime (FEP 30 μg), cefepime/clavulanic acid (FEP 30 μg), ceftazidime (CAZ 30 μg), ceftazidime/clavulanic acid (CAC 30/10 μg) cefotaxime (CTX 30 μg), cefotaxime/clavulanic acid (CEC 30/10 μg), and cefoxitin (FOX 30 μg) were used. An increase of ≥5 mm in the zone diameter of a combination of the antibiotic with clavulanic acid against the zone diameter of the antibiotic alone was reported as corresponding to an ESBL phenotype. The isolates were characterized as AmpC if resistance to cefotaxime and ceftazidime was recorded without induction by clavulanic acid and exhibited a ≥5 mm increase in the inhibition zone. For quality control, the type cultures *E. coli* ATCC 25922 and *K. pneumoniae* ATCC 700603 were used.

### 2.5. PCR Screening of Isolates 

For the determination of the genotype coding the ESBL or AmpC phenotypes, a multiplex PCR method was employed, as described by Fang et al. [18]. The method is suitable for detecting the presence of the *bla*_TEM_, *bla*_SHV_, *bla*_CTX-M,_ and *bla*_OXA_ genes in one multiplex PCR reaction. In brief, pure cultures of the isolates were subjected to DNA extraction, according to Tsiouris et al. [19]. One loopful of cells was dispersed in 100 μL dispersal buffer (50 mM Tris-HCl, 50 mM ethylenediaminetetraacetate (EDTA), 1% *v*/*v* Triton X-100, pH 7.5), in which 100 μL of lysis buffer I [50 mM Tris-HCl, 50 mM EDTA, 4 M guanidine hydrochloride (GuHCl), 10 mM CaCl_2_, 1% *v*/*v* Triton X-100, 2% N-Lauroyl-Sarcosine, pH 7.5], and 25 μL of proteinase K solution (22.4 mg/mL) were added, followed by incubation at 56 °C for 1 h. Consequently, 250 μL of lysis buffer II (50 mM Tris-HCl, 25 mM EDTA, 8 M GuHCl, 3% *v*/*v* Triton X-100, 3% N-Lauroyl-Sarcosine, pH 6.3) were added, and the mixture was incubated at 70 °C for 10 min. All reagents used for DNA extraction were manufactured by AppliChem GmbH (Darmstadt, Germany). After incubation, 250 μL of absolute ethanol was added, and the mixture was applied to silica columns (Qiagen, Venlo, The Netherlands). The column was then washed twice with wash I buffer [25 mM Tris-HCl, 4 M GuHCl, 50% ethanol (Merck GmbH, Darmstadt, Germany), pH 6.6] and once with wash buffer IΙ (10 mM Tris-HCl, 80% ethanol, 100 mM NaCl, pH 6.6). The DNA was eluted with ultrapure water (AppliChem GmbH, Darmstadt, Germany) and stored at −30 °C until examination. DNA quality and recovery were evaluated with the use of a NanoDrop microvolume spectrophotometer (Nanodrop 2000, Thermo Fisher Scientific, Waltham, MA, USA).

PCR was performed at 25 μL volume containing 5 μL of 5× OneTaq Standard Reaction Buffer (NEB), 0.5 μL of DNTP mix (NEB), 1 U of OneTaq ™ Hot Start DNA Polymerase (New England Biolabs, Hitchin, UK) and 2 μL of the sample. The primers used are shown in Table 1. The reaction volume was made up to 25 μL with the addition of sterile MiliQ grade water. PCR was performed in a thermal cycler (LabCycler gradient, SensoQuest, Göttingen, Germany). Initial denaturation (30 s at 95 °C) was followed by 30 cycles of amplification (denaturation at 94 °C for 30 s, annealing at 62 °C for 90 s, and extension at 72 °C for 60 s), ending with a final extension at 72 °C for 10 min. The PCR products were visualized in 1.5% agarose gels stained with ethidium bromide with the use of a UVP DigiDoc-It^®^ 125 gel imaging system (UVP, Cambridge, UK). 

### 2.6. Statistical Analysis 

Frequencies were compared after the application of contingency tables and the use of χ^2^ goodness-of-fit tests with the use of the IBM^®^ SPSS^®^ Statistics software (version 25). The level of significance was set at 5% (critical *p*-value: 0.05).

## 3. Results

**Figure 1:**
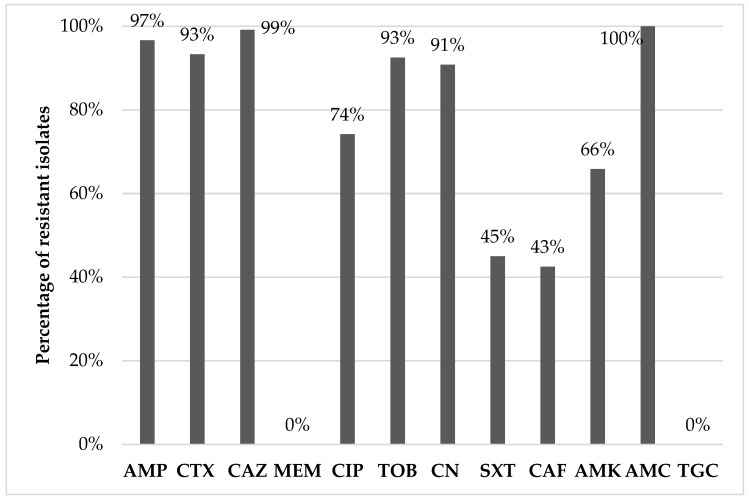
Percentage of AMR of the *Escherichia coli* isolates (AK: amikacin; AMC: amoxicillin-clavulanic acid; AMP: ampicillin; CAF: chloramphenicol; CAZ: ceftazidime; CIP: ciprofloxacin; CN: gentamicin; CTX: cefotaxime; MEM: meropenem; SXT: trimethoprim-sulfamethoxazole; TGC: tigecycline. TOB: tobramycin).

Of the 100 pooled samples tested, 64 (64%) were positive for ESBL isolates. In total, 120 *E. coli* isolates were recovered. The resistance phenotypes, the β-lactamase production phenotype, and the genetic background of β-lactam resistance are presented in Table 2. The percentages of antibiotic resistance among the isolates ranged from 0% (against MEM or TGC) to 100% (against AMC), as shown in Figure 1. According to the disc diffusion method, all isolates were multi-drug resistant (MDR) since they were resistant to at least three classes of antibiotics (Table 1). The most common resistance phenotype featured resistance to AMP, CTX, CAZ, CIP, TOB, CN, SXT, AMK, and AMC and was shared among 15% (*n* = 18) of the isolates examined. The resistance phenotype [AMP, CTX, CAZ, CIP, TOB, CN, AMK, AMC] was shared among 10% (*n* = 12) of the isolates, whereas the resistance phenotypes [AMP, CTX, CAZ, CIP, TOB, CN, SXT, CAF, AMK, AMC] and [AMP, CTX, CAZ, CIP, TOB, CN, SXT, AMC] were both found in 9.17% (*n* = 11) of the isolates.

**Figure 2:**
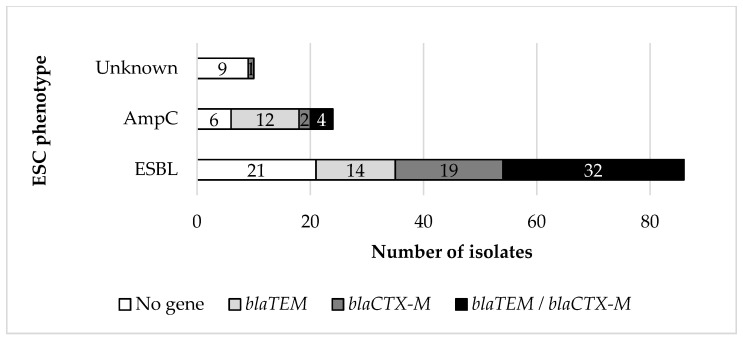
Detection of ESBL encoding genes related to the ESBL phenotype.

Concerning the phenotype of β-lactam resistance, 86 (71.67%) and 24 (20%) of the 120 isolates were classified as true ESBL- or AmpC-producers, respectively, whereas 10 of the isolates (8.33%) were not phenotypically categorized as β-lactamase producers by the classification scheme used [significant difference in the occurrence of phenotypes; χ^2^(2) = 81.800, *p* < 0.001]. Concerning the occurrence of the investigated β-lactamase genes (*bla*_TEM_, *bla*_SHV_, *bla*_CTX-M_, and *bla*_OXA_), *bla*_TEM_ was detected in 26 (21.67%) isolates, *bla*_CTX-M_ was detected in 22 (18.66%) isolates, whereas 36 (30.00%) isolates harbored both *bla*_TEM_ and *bla*_CTX-M_ genes (Figure 2). No isolate harboring *bla*_SHV_ or *bla*_OXA_ was detected. Among the isolates of this study that displayed an ESBL phenotype and harbored *bla* determinants, 14 harbored *bla*_TEM_ genes, 19 harbored *bla*_CTX-M_ genes, whereas 32 harbored both *bla*_TEM_ and *bla*_CTX-M_ genes. The frequency of occurrence of these three categories was not statistically different [χ^2^(2) = 3.271, *p* = 0.195]. In contrast, among the AmpC phenotype isolates, the ones harboring both *bla*_TEM_ and *bla*_CTX-M_ genes (*n* = 4) were fewer than those harboring *bla*_CTX-M_ genes (*n* = 12) only [χ^2^(1) = 4.000, *p* = 0.046]. Overall, a significant interaction between the ESBL or AmpC phenotype and the occurrence of *bla* genes was observed [χ^2^(2) = 13.364, *p* = 0.001] with ESBL-phenotype isolates carrying significantly more frequently the [*bla*_TEM_ + *bla*_CTX-M_] combination than the AmpC-phenotype isolates.

Regarding the ESC phenotype, overall, 36 isolates (30%) harbored both *bla*_TEM_ and *bla*_CTX-M_ genes, 26 (21.67%) harbored *bla*_TEM_ genes, and 22 (18.33%) harbored *bla*_CTX-M_ genes. In 6 isolates characterized phenotypically as true ESBL producers, no β-lactamase encoding genes were detected. Among the isolates exhibiting an AmpC phenotype, 12 isolates (50%) harbored *bla*_TEM_ genes, and four (16.67%) both *bla*_TEM_ and *bla*_CTX-M_ genes. In six AmpC phenotype isolates (25.00%), no *bla*_TEM_, *bla*_SHV_, *bla*_CTX-M_, or *bla*_OXA_ genes were detected. The ESBL- or AmpC-phenotype isolates in which no β-lactamase encoding genes were detected most frequently exhibited the [AMP, CTX, CAZ, CIP, TOB, CN, CAF, AMC] (ESBL = 44, 44%, *n* = 4), [AMP, CTX, CAZ, TOB, CN, AMC] (ESBL = 33.33%, AmpC = 50%, *n* = 5), [AMP, CAZ, CIP, TOB, CN, CAF, AMC] (ESBL = 100%, *n* = 2), and [AMP, CAZ, TOB, CN, CAF, AMC] (ESBL = 100%, *n* = 2) phenotypes.

## 4. Discussion

Poultry meat (mostly of chicken origin, but also derived from turkey and other avian species) is an easily accessible, low-cost protein source and a widely consumed staple food throughout the world, both in high-income and in low- and middle-income countries. Production, processing, and trade of poultry meat are inherently associated with a significant risk of contamination by bacteria, as well as by AMR determinants [20]. A point of grave concern is the dissemination and acquisition of food-borne bacteria that express AMR (especially multiple-drug resistance, MDR) against critically important antibiotics (i.e., 3rd, 4th, and 5th generation cephalosporins, glycopeptides, macrolides and ketolides, polymyxins, and [fluoro]quinolones) in human medicine [21,22], since food-producing animals and foods of animal origin are well-established reservoirs for AMR bacteria and genes [14,23,24]. Specifically, bacteria resistant to extended-spectrum cephalosporins have been identified as a significant zoonotic hazard, as they are increasingly isolated in animals and humans and are often implicated in infections for which the chemotherapeutic options are limited [23,25,26,27]. Resistance to extended-spectrum cephalosporins in Enterobacterales is typically conferred by ESBL, AmpC, or carbapenemase enzymes [24,28,29]. ESBL-producing Enterobacterales have historically been more prevalent in Europe, whereas AmpC enzymes are also a serious concern, especially in southern America [23,25,30]. Both seem to have been detected in virtually every stage of chicken or turkey meat production (from farm to slaughterhouses and in the final retail), much like the undoubtedly most common Enterobacterales species, i.e., *E. coli* [21,22], with chicken and turkeys considered as a reservoir of extended-spectrum cephalosporin-resistant *Enterobacteriaceae* [14,31]. 

The presence of extended-spectrum cephalosporin-resistant *E. coli* in the present study was detected in 64% of the pooled poultry skin samples, a percentage that lies well within the wide range reported during the last decade in several studies performed in other European studies. Xexaki et al. [32] have studied the prevalence of antibiotic-resistant *E. coli* in farmed broilers in Greece, essentially from the same area as the present study; they report a lower prevalence of ESC-producing *E. coli* with 13.6% of the isolates producing ESBL and 2.7% producing AmpC β-lactamase. The lower percentages can be attributed to the methodology used that did not select ESBL-producing microorganisms or to a further step in poultry processing that contaminates meat along the production chain. Concerning poultry meat, Randall et al. [33] examined chicken meat samples in the United Kingdom collected in 2016 and 2018 and reported a respective 13.6% and 45% detection rate of ESBL and/or AmpC phenotype *E. coli*. Huizinga et al. [34] reported a 54.3% prevalence of ESBL-producing *Enterobacteriaceae* (94.4% of which were identified as *E. coli*) in chicken meat samples analyzed between 2014 and 2015 in the Netherlands. In a study conducted in Bosnia and Herzegovina, Hadžić-Hasanović et al. [35] isolated ESBL-producing *E. coli* in 29 out of the 100 chicken skin samples collected in 2018–2019, whereas Egervärn et al. [14] reported the isolation of ESBL/pAmpC *E. coli* in 34 out of 90 broiler meat samples (37.8%) imported into Sweden from across Europe in 2010–2011. On the other side of the spectrum, extended-spectrum cephalosporin-resistant (ESBL- and/or AmpC-phenotype) *E. coli* detection rates as high as 71.9–93.3% were reported in chicken (and turkey) meat samples in countries such as Spain, Germany, France, etc. [24,25,28,36]. Specifically, Egea et al. [28] report the rise in the prevalence of ESBL-producing *E. coli* in chicken meat samples from Spain from 62.5% in 2007 to 93.3% in 2010. Casella et al. [25] report that 91.7% of the 48 chicken samples from France tested positive for ESBL-producing *E. coli*. Kaesbohrer et al. [36] have examined several types of meat for the occurrence of ESBL-producing *E. coli*; they report that cefotaxime resistance was most common in *E. coli* isolated from chicken meat (74.9%). 

Among the investigated isolates, resistance to aminoglycosides (tobramycin, gentamicin, and amikacin) ranged from 65.8% (79/120 for amikacin) to 92.5% (111/120 for tobramycin). A total of 74.2% (110/120) of the isolates were resistant to ciprofloxacin (used as an indicator fluoroquinolone), whereas 45% (54/120) and 41.7% (50/120) were also resistant to trimethoprim-sulfamethoxazole and chloramphenicol. All isolates were susceptible to meropenem and tigecycline, two of the last-resort antibiotics in human (and occasionally in companion animals) medicine. Interestingly, concurrent resistance yielded an MDR rate of 100% since all 120 isolates were resistant to antibiotics belonging to at least three classes. This finding was barely surprising as extended-spectrum cephalosporin-resistant *E. coli* isolates have been regularly known to express MDR patterns [20,23,28]. Díaz-Jiménez et al. [24] examined 100 chicken and turkey breast samples in 2016–2017 and found that 136 out of the 137 *E. coli* isolates were MDR, whereas Egervärn et al. [14], Moawad et al. [29], Nüesch-Inderbinen et al. [26], and Irrgang et al. [30] also reported very high MDR rates in extended-spectrum cephalosporin-resistant *E. coli* isolated from chicken and turkey samples, as well as from poultry-containing raw meat-based diets. Particularly, resistance to fluoroquinolones, aminoglycosides, and trimethoprim-sulfonamide combinations is very frequent in MDR ESBL/AmpC-producing isolates and raises profound concerns about its implication in the treatment of severe infections in both animals and humans [14,34,35]. In general, AMR in *E. coli* isolated from poultry is more prominent compared with other food-producing species [14,24,29], with a trend for turkeys to harbor resistant bacteria more frequently than broilers, possibly due to increased antibiotic use and/or prolonged fattening [21,24]; still, the latter finding is controversial, since data on an increased AMR occurrence in chicken compared with turkeys have also been published [36]. Concerning the AMR patterns observed, most of them correlate with the treatments usually followed for the most common poultry diseases. Specifically, the proposed antibiotics used for *E. coli* infections are aminopenicillins, fluoroquinolones, aminoglycosides, sulfonamides/trimethoprim, or tetracyclines [37]. Unfortunately, no data exist on the use of antibiotics in poultry, specifically in Greece. However, according to the sales of veterinary antimicrobial agents in veterinary medicine in Greece in 2021 [38], the most commonly used antibiotics in food-producing animals are tetracyclines, penicillins, aminoglycosides, sulfonamides, fluoroquinolones, macrolides and amphenicols (sales of veterinary antimicrobial agents in 31 European countries in 2021); the high resistance rate observed for β-lactams and aminoglycosides in the present study could possibly be due to the extensive use of these agents.

The increased prevalence of ESBL compared to the AmpC phenotype observed in this study (86/120 vs. 24/120 isolates) is on par with available data published in Europe. For example, Casella et al. [25] examined 77 non-clonal *E. coli* isolates from chicken meat sampled in France, of which 74 and three displayed ESBL and AmpC phenotypes, respectively. Moreover, Muller et al. [23] reported ESBL and AmpC phenotypes in 29 and seven *E. coli* isolates from imported meat in Germany. However, Egervärn et al. [14] reported a similar prevalence (18.9%, 90 samples) of ESBL- and AmpC-producing *E. coli* in broiler meat samples imported into Sweden (with a remarkable, complete prevalence of AmpC phenotype in the samples of Danish origin). Conversely, Kaesbohrer et al. [36] reported a higher prevalence of the AmpC-encoding *bla_CMY_*_-2_ gene in 138 *E. coli* isolated from chicken meat samples compared to classic ESBL genes (such as *bla*_TEM_, *bla*_SHV,_ and *bla*_CTX-M_) prevalence; still, this involved the genotype of the isolates and not their phenotypic expression. 

Rather surprisingly, no *bla*_SHV_ (or *bla*_OXA_) genes were detected, although they are frequently observed in human patients in Greece [39]. Poultry, in general, are a reservoir of *bla*_SHV-12_, *bla*_SHV-2_, *bla*_SHV-2a,_ and other variants of this category [30,32,33]. Especially *bla*_SHV-12_ has been very widespread in ESBL-producing *E. coli* isolated from chicken and/or turkey meat samples and has occasionally been found to be the most prominent ESBL gene [24,28,33]. However, it seems that *bla*_CTX-M_ genes (mainly *bla*_CTX-M-1_, *bla*_CTX-M-14,_ and related variants) have lately gained momentum, and, in many cases, their occurrence is now considered to have surpassed the one of *bla*_SHV_ not only in poultry but in livestock in general [14,22,26]. Indeed, bla_CTX-M-1_ has been shown to constitute the principal ESBL enzyme in several studies on extended-spectrum cephalosporin-resistant *E. coli* of chicken and turkey origin, with other variants, such as *bla*_CTX-M-15_ (most prevalent in humans), *bla*_CTX-M-2_, *bla*_CTX-M-8_, *bla*_CTX-M-9_, *bla*_CTX-M-12_, *bla*_CTX-M-14/17_, *bla*_CTX-M-32_, *bla*_CTX-M-79,_ or *bla*_CTX-M-104_ also being seldomly detected [25,26,31,33,34,35]. Concerning *bla*_TEM_ genes (detected in more than half of the isolates in this study), they seem to represent the third most prevalent category in ESBL-producing *E. coli* isolates of poultry origin. Overall, the *bla*_TEM-52_, *bla*_TEM-52B/C_, *bla*_TEM-104_, and *bla*_TEM-135_ variants have been found to be responsible for the production of extended-spectrum cephalosporin-inactivating enzymes, at least in studies performed in Europe [14,24,25,29,34]. Finally, *bla*_OXA_ genes are rather rarely detected in *E. coli* of avian origin [29], and their absence in the isolates of the present study does not likely pose any new concerns.

To our knowledge, the present study is the first to report the presence of extended-spectrum cephalosporin-resistant *E. coli* isolates in retail poultry in Greece, also exploring their genetic context. This information can elucidate the current situation and provide a starting point for the evaluation of time trends to assess the need for the implementation of intervention measures. Further investigation is required to establish a sound connection between the presence of ESC-producing *E. coli* in food and their impact on public health.

## 5. Conclusions

The increased prevalence of extended-spectrum cephalosporin-resistant *E. coli* in poultry (especially chicken meat) raises both public health and healthcare cost-associated concerns. AMR bacteria, in general, and extended-spectrum cephalosporin-resistant *E. coli* likely rise from overuse/misuse of antibiotics in livestock or from contamination along the production/storage chain. Increased prevalence of isolates with an ESBL and/or an AmpC-phenotype, mostly multi-drug resistant, was observed in the present study, therefore indicating that poultry marketed in Greece can be a vector for transmission of resistance factors to humans. Still, their occurrence was within the range reported in other European countries. The genetic background of the isolates for ESBL production featured the presence of variants of the *bla*_CTX-M_ and the *bla*_TEM_ genes, although non-gene harboring isolates were also isolated; no *bla*_SHV_ or *bla*_OXA_ genes were detected. Further studies are needed in order to access microbial resistance trends, elucidate possible transmission routes and further strengthen public health surveillance.

## Figures and Tables

**Table 1:** Characteristics of the primers used for the detection of ESBL or AmpC genes.

Target	Primer Sequence (5′ to 3′)	Size (bp)
*bla*_TEM_ genes	CGC CGC ATA CAC TAT TCT CAG AAT GA	445
	ACG CTC ACC GGC TCC AGA TTT AT	
*bla*_SHV_ genes	CTT TAT CGG CCC TCA CTC AA	237
	AGG TGC TCA TCA TGG GAA AG	
*bla*_CTX-M_ genes	ATG TGC AGY ACC AGT AAR GTK ATG GC	593
	TGG GTR AAR TAR GTS ACC AGA AYC AGC GG	
*bla*_OXA_ genes	ACA CAA TAC ATA TCA ACT TCG C	813
	AGT GTG TTT AGA ATG GTG ATC	

**Table 2:** Resistance profiles, β-lactam resistance phenotypes, and genetic determinants of the isolates examined for their antimicrobial resistance.

No. of Antibiotics against Which Resistance Was Detected	No. of Isolates	Resistance Phenotype	β-Lactam Resistance Phenotype			ESBL Gene Class		
			ESBL	AmpC	Unknown	*bla*_TEM_	*bla*_CTX-M_	*bla*_TEM_/*bla*_CTX-M_
9	18	AMP, CTX, CAZ, CIP, TOB, CN, SXT, AMK, AMC	12	4	2	3	3	9
8	12	AMP, CTX, CAZ, CIP, TOB, CN, AMK, AMC	8	2	2	3	-	6
10	11	AMP, CTX, CAZ, CIP, TOB, CN, SXT, CAF, AMK, AMC	8	2	1	3	2	6
8	11	AMP, CTX, CAZ, CIP, TOB, CN, SXT, AMC	9	2	-	5	2	3
9	9	AMP, CTX, CAZ, CIP, TOB, CN, CAF, AMK, AMC	7	2	-	2	3	2
8	9	AMP, CTX, CAZ, CIP, TOB, CN, CAF, AMC	5	2	2	3	-	-
7	9	AMP, CTX, CAZ, TOB, CN, AMK, AMC	7	-	2	-	5	2
8	8	AMP, CTX, CAZ, TOB, CN, CAF, AMK, AMC	6	2	-	-	3	2
6	6	AMP, CTX, CAZ, TOB, CN, AMC	3	3	-	-	2	-
8	3	AMP, CAZ, CIP, TOB, CN, SXT, AMK, AMC	2	1	-	2	-	-
9	2	AMP, CTX, CAZ, MEM, CIP, TOB, SXT, AMK, AMC	2	-	-	-	-	2
7	2	AMP, CTX, CAZ, CN, CAF, AMK, AMC	2	-	-	-	-	-
8	2	AMP, CTX, CAZ, CIP, TOB, SXT, CAF, AMC	2	-	-	-	2	-
8	2	CTX, CAZ, CIP, TOB, SXT, CAF, AMK, AMC	2	-	-	-	-	2
9	1	AMP, CTX, CAZ, CIP, TOB, CN, SXT, CAF, AMC	-	1	-	-	-	-
7	2	AMP, CTX, CAZ, CIP, CN, AMK, AMC	2	-	-	-	-	2
7	1	AMP, CTX, CAZ, CIP, CN, CAF, AMC	-	1	-	-	-	-
6	2	AMP, CAZ, TOB, CN, CAF, AMC	2	-	-	-	-	-
6	1	AMP, CTX, CAZ, CIP, CN, AMC	-	1	-	1	-	-
6	2	AMP, CTX, CAZ, CIP, SXT, AMC	2	-	-	2	-	-
6	2	AMP, CTX, CAZ, TOB, SXT, AMC	2	-	-	2	-	-
7	2	AMP, CAZ, CIP, TOB, CN, CAF, AMC	2	-	-	-	-	-
7	1	AMP, CTX, CAZ, CIP, TOB, CN, AMC	-	1	-	-	-	-
7	1	AMP, CTX, CAZ, TOB, CN, CAF, AMC	-	-	1	-	-	-
4	1	AMP, CAF, AMK, AMC	1	-	-	-	1	-
	120		86	24	10	26	23	36

AK: amikacin; AMC: amoxicillin-clavulanic acid; AMP: ampicillin; CAF: chloramphenicol; CAZ: ceftazidime; CIP: ciprofloxacin; CN: gentamicin; CTX: cefotaxime; MEM: meropenem; SXT: trimethoprim-sulfamethoxazole; TGC: tigecycline. TOB: tobramycin.

## Data Availability

The data presented in this study are available upon request from the corresponding author.
